# Comparison of cricket diet with peanut-based and milk-based diets in the recovery from protein malnutrition in mice and the impact on growth, metabolism and immune function

**DOI:** 10.1371/journal.pone.0234559

**Published:** 2020-06-11

**Authors:** Rachel S. Bergmans, Maria Nikodemova, Valerie J. Stull, Ashley Rapp, Kristen M. C. Malecki

**Affiliations:** 1 Department of Psychiatry, University of Michigan, Ann Arbor, Michigan, United States of America; 2 Department of Population Health Sciences, University of Wisconsin-Madison, Madison, Wisconsin, United States of America; 3 Global Health Institute, University of Wisconsin-Madison, Madison, Wisconsin, United States of America; 4 Department of Epidemiology, University of Michigan, Ann Arbor, Michigan, United States of America; National Institute for Agronomic Research, FRANCE

## Abstract

Some evidence suggests that edible insects could be used to treat malnutrition following protein deficiency. However, additional studies are needed to better assess the potential of edible insects as a therapeutic food supplement and their long-term impact on recovery from malnutrition. The goals of this study were to investigate the effectiveness of a cricket-based diet in recovery from protein-malnutrition in early life, and to compare cricket protein to more traditional sources used for food fortification and supplementation. Protein-malnutrition was induced by administration of an isocaloric hypoprotein diet (5% protein calories) in young male mice for two weeks during puberty, followed by a six-week recovery period using a cricket-, peanut- or milk-based diet. We examined the impact of protein-malnutrition and subsequent recovery on body weight, growth and select biomarkers of inflammation and metabolism. Protein-malnutrition resulted in growth retardation, downregulation of inflammatory markers in spleen tissue, decreased levels of serum triglycerides, and elevated serum levels of leptin and adiponectin. The cricket-based diet performed equally well as the peanut- and milk-based diets in body weight recovery, but there were differences in immune and metabolic markers among the different recovery diets. Results suggest edible crickets may provide an alternative nutrient-dense protein source with relatively low environmental demands for combating the effects of early-life malnutrition compared to more traditional supplementation and fortification sources. Additional investigations are needed to examine the short and long term impacts of different recovery diets on metabolism and immune function.

## Introduction

Malnutrition is a global health problem, especially among children. Over 5.9 million children die annually due to malnutrition before their fifth birthday [1]. Malnutrition occurs in many forms, from acute to chronic, and results from either insufficient caloric intake or insufficient intake of select essential nutrients [2]. Undernutrition due to a lack of micronutrients and macronutrients, including protein, increases the risk of immune deficiency [3]. This is a significant concern because infectious diseases are a leading cause of mortality among children living in resource-poor countries [4]. Even in less severe cases, malnutrition can lead to serious health consequences that persist over the life course [5, 6]. For example, early life malnutrition is associated with cognitive deficits, obesity, type 2 diabetes and cardiovascular diseases in adulthood [7–9]. Global malnutrition is a growing concern due to interactions between environmental, economic and sociopolitical factors that affect the rapidly changing landscape of regional and local food systems [10]. While much progress has been made in reducing protein-malnutrition, significant disparities exist on a global scale [10]. Moreover, the sustainability of food systems worldwide faces pressure from increasing demands for protein, rising food costs and climate change [11].

Novel solutions to alleviate global malnutrition are needed beyond conventional food supplementation and fortification approaches [12–14]. Current malnutrition interventions primarily rely on fortified flours or supplements with a high lipid content. While fortified flours provide access to additional caloric energy, they are typically made from inexpensive sources (e.g., cereals, legumes) that are not ideal for providing a rapid response to malnutrition [15]. The optimal nutritional characteristics for treating malnutrition are not fully known, but protein is a key factor—not only its quantity, but also its quality and digestibility [16–18]. Conventional livestock are often considered an ideal source of protein; however, they can be difficult to produce in many places and expensive due to the resources required to rear them [19].

The United Nations Food and Agricultural Organization, among others, has proposed that edible insects could provide an alternative source to support efforts to combat global food insecurity [20–22]. Edible insects are increasingly considered a practical, nutrient-dense protein source with relatively low environmental demands [23–25]. For example, some edible insects can be reared on agricultural by-products that would not otherwise be put to use [26]. Additionally, producing edible crickets requires less water, feed and space than conventional livestock, while providing an equal amount of protein per kilogram [27]. Furthermore, thousands of edible insect species are already incorporated in diets across the globe, and are consumed by populations across the globe, including by groups in Southern Africa where rates of malnutrition are disproportionally high [20, 28, 29]. Insects are a good source of protein, essential amino-acids and micronutrients, which are often lacking in carbohydrate-based diets [24, 30, 31]. Some evidence suggests that edible insects could be used to treat malnutrition following protein deficiency [32]. However, additional studies are needed to better assess the potential of edible insects as a therapeutic food supplement and the long-term impacts of insects on recovery from malnutrition.

Using a mouse model, the goal of this study was to investigate the effectiveness of using a cricket-based diet to recover from protein-malnutrition in early life, and to assess how it compares to protein sources traditionally used for food fortification and supplementation. Protein-malnutrition was induced by administration of an isocaloric hypoprotein diet in young male mice for two weeks during puberty, followed by a six-week recovery period on a cricket-, peanut- or milk-based diet. We examined the impact of protein-malnutrition and subsequent recovery on body weight, growth and select biomarkers of inflammation and metabolism.

## Material and methods

### Animals

Three-week-old male mice (n = 65) were purchased from Charles River CD1-IGS (Wilmington, MA, USA). Animals were housed in AAA-LAC accredited facilities under standard conditions (12-hour light/dark cycle, food and water available *ad libitum*). All experiments were conducted under protocol M005599 approved by the University of Wisconsin Institutional Animal Care and Use Committee. After a 10-day acclimatization period, animals were divided into experimental groups (n = 10–12). Animals’ wellbeing was monitored daily and body weight was recorded three times per week. At the end of experiments, animals were euthanized by the overdose of isoflurane followed by collection of blood via cardiac puncture and collection of spleen tissue. Blood was allowed to clot for 30–40 min at room temperature followed by centrifugation for 10 minutes. Serum was immediately transferred to cryovials and frozen at -80°C until further use. Spleen tissues were frozen immediately on dry ice and stored at -80°C until further use. All animals were sacrificed between 11am and 1pm to minimize differences in serum leptin and adiponectin due to diurnal rhythm [33–35].

### Diet composition and regime

#### Diet composition

A total of five irradiated diets manufactured by Envigo (Madison, USA) were used throughout the study: an initial weaning diet (2020 diet); a hypoprotein diet; standard adult diet (2018 diet); and, 3 intervention diets (milk, peanut, cricket protein). Table 1 presents the specific composition and caloric content for each diet. All diets had a similar energy density ranging from 3.1 to 3.7 kcal/g. The 2020 weaning diet was used on all pups and was formulated to include 24% protein with wheat and cornmeal (soy protein-free). The hypoprotein diet used to induce malnutrition was isocaloric and contained only 5% protein derived from corn gluten meal. Compared to the standard diet, the hypoprotein diet also had reduced calories from fat and increased calories from carbohydrates in order to keep the diet isocaloric. A control diet (2018) containing wheat, corn gluten meal and soybean meal served as a standard adult mouse diet. The three intervention diets used in the recovery phase were designed by a nutritionist at the Teklad Diets Envigo to vary based on primary protein sources inlcuding 1) cricket (*Gryllodes sigillatus*), 2) cow skim milk powder and 3) peanut flour. For the cricket diet, ready-to-use cricket powder was obtained from Entomo Farms (Norwood, Canada). For the peanut diet, peanut flour was obtained from Golden Peanut and Tree Nuts (Alpharetta, USA). Envigo provided the cow milk powder. The three intervention diets were formulated to meet minimum mouse dietary needs for minerals and vitamins.

**Table 1:** Diet composition.

	Weaning diet	Standard adult Diet		Intervention—Recovery diets		
	2020	2018	Hypoprotein	Cricket	Milk	Peanut
Proteins (% calories)	24	24	5.0	19.8	21.3	21.0
Carbohydrates (% calories)	60.0	58	84.2	56.1	59.9	59.7
Fat (% calories)	16.0	18	10.8	24.1	19.8	19.3
Energy density						
kcal/g	3.1	3.1	3.5	3.7	3.6	3.5
kJ/g	13.0	13.0	14.6	15.5	15.1	14.6

#### Diet regime

**Fig 1:**
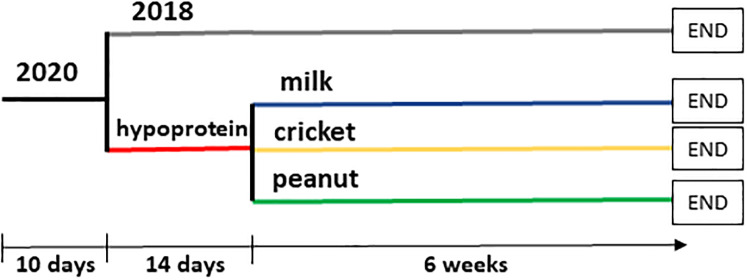
Experimental design. At the age of 3 weeks, male mice started on the 2020 diet for 10 days. After then, animals were divided into control and low protein diet groups. Control group received 2018 diet (control) whereas animals in the hypoprotein group received low protein diet for 2 weeks. After this period, a portion of mice from each group was euthanized for tissue harvest. Remaining mice in the control group continued on 2018 diet. Mice in hypoprotein group were divided into three groups receiving intervention diets with different source of protein (either milk, peanut or cricket). After 6 weeks on the control or intervention diets, mice were euthanized followed by a tissue harvest.

Fig 1 illustrates the three phases of the diet regime. All mice were weaned (phase 1) for a period of ten days using the 2020 control diet. Protein-malnutrition was induced in three experimental groups for two weeks using the hypoprotein diet (phase 2). A normal control group fed the 2018 diet was also included to model healthy growth and development without protein malnutrition. At the end of two weeks, a portion of mice from all groups was euthanized for tissue harvest. For the next six weeks (phase 3), mice previously fed the hypoportein diet were assigned to one of three protein-based recovery diets (i.e. cricket, milk, or peanut) and the control group continued to receive the 2018 diet. All mice were then euthanized for tissue harvest.

### Triglyceride and adipokine assays

Serum triglycerides levels were analyzed using EnzyChrom Triglyceride Assay kit (BioAssay Systems). Serum samples were diluted 1:5 with water and assayed in duplicates according to manufacturer’s protocol. Results were calculated from an 8-point standard curve.

Serum leptin and adiponectin were analyzed using Quantikine ELISAs (both from R&D Systems). Samples for leptin assay were diluted 20x with RD5-3 buffer provided in the kit. Samples for adiponectin assay were diluted 2000x with RD2-26 buffer provided in the kit. All samples were assayed in duplicates and results were calculated from 8-point standard curves.

### RNA extraction and qRT-PCR

To determine gene expression for TLR4, TNFα, IL-1β, IFNγ and IL-4 total RNA was extracted from the spleens for a subset of mice from each diet group using Tri-Reagents (Sigma) according to the manufacturer’s protocol. For the PCR analysis, total RNA was first transcribed to cDNA using MMLV reverse transcriptase kit (Invitrogen) according to the manufacturer’s protocol followed by quantitative PCR using SYBR green master solution (Applied Biosystems). The PCR was run on QuantStudio 7 Flex for 40 cycles. Primer sequences are provided in Table 2. Results were normalized to 18S levels and relative gene expression was calculated using the ΔΔC_t_ method [36]. Data are expressed as fold change relative to the control diet group.

**Table 2:** Primer sequences for PCR.

Primer sequences	
18S	F: 5′-CGG GTG CTC TTA GCT GAG TGT CCC G-3′
	R: 5′-CTC GGG CCT GCT TTG AAC AC-3′
INFγ	F: 5’-TGG CAT AGA TGT GGA AGA AAA GAG-3’
	R: 5’-TGC AGG ATT TTC ATG TCA CCA T-3’
IL-1β	F: 5’-TCA AAG TGC CAG TGA ACC CC-3’
	R: 5’-GGT CAC AGC CAG TCC TCT TAC-3’
IL-4	F: 5’-CTC GAA TGT ACC AGG AGC CA-3’
	R: 5’- GTG GTG TTC TTC GTT GCT GTG-3’
TLR4	F: 5’-GAG GCA GCA GGT GGA ATT GTA T-3’
	R: 5’-TTC GAG GCT TTT CCA TCC AA-3’
TNFα	F: 5′- TGT AGC CCA CGT CGT AGC AA-3′
	R: 5′- AGG TAC AAC CCA TCG GCT GG-3′

### Statistical analysis

Two-way ANOVA followed by Holm-Sidak test was used to determine statistically significant differences in body weight across time and between different diets. Serum biomarkers and mRNA levels were analyzed by one-way ANOVA followed by Dunn’s test when appropriate. All results are expressed as means ± SE. Differences between groups were considered statistically significant if p< 0.05. Number of animals/group is indicated in figure legends.

## Results

### Body weight and growth

#### Protein-malnutrition

**Fig 2:**
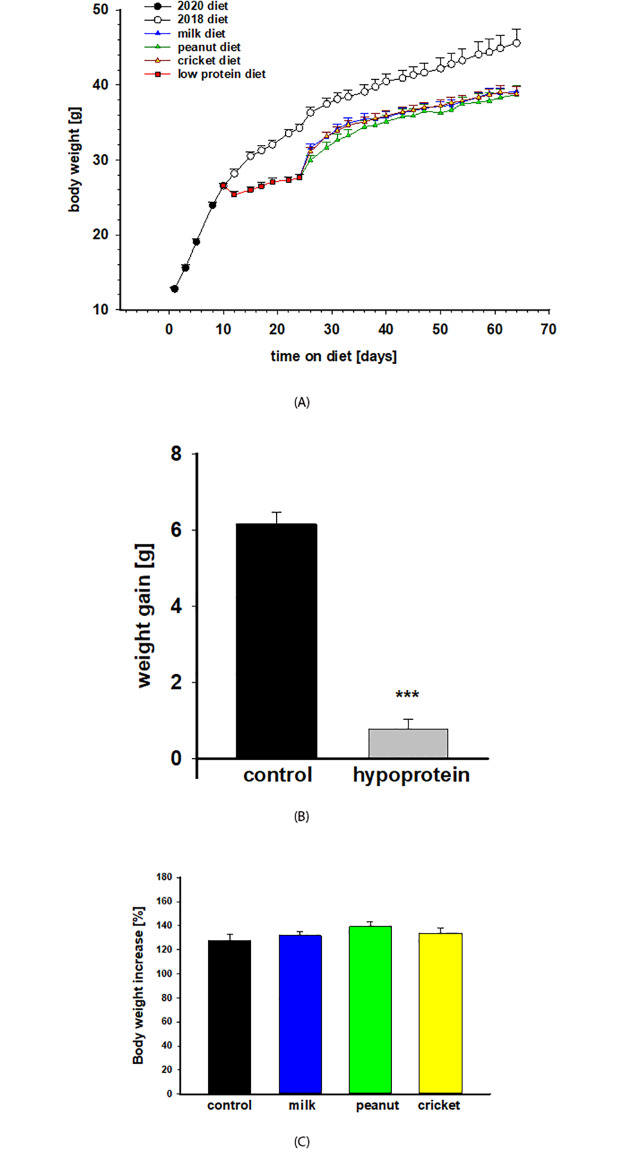
Body weight and growth. **A)** Body weight was measured three times per week throughout the experiment. Mice were on 2020 weaning diet starting at age of 3 weeks for 10 days. Control mice group continued on 2018 diet, whereas mice in experimental group were put on hypoprotein diet for 2 weeks. Next, three intervention diets (milk, peanut and cricket protein) were administered for 6 weeks. Two-way ANOVA revealed significant differences between control and hypoprotein groups during a two-week period and control and intervention groups during the 6-week period. There were no statistically significant differences among interventions groups. N = 12/group. **B)** Body weight of the control group on 2018 diet and low protein group after two weeks of malnutrition. **C)** Percentage of body weight increase during 6-week period from the beginning of intervention diet after malnutrition (100%) to the end of the experiment. Results are expressed as means ± SE. *** p< 0.001.

Protein-malnutrition induced by an isocaloric hypoprotein diet (5% protein calories) administered for two weeks in young male mice resulted in growth retardation but without body weight loss (Fig 2A). Mice on the 2018 control diet gained on average 6.2 grams, representing a 24% increase in their body weight, during the 2-week period (Fig 2B). In contrast, mice on low protein diet gained only 0.7 g in the same time period, corresponding to less than a 2% increase in their body weight (Fig 2B). After 2 weeks, malnourished mice had significantly lower body weight (28.9 g) compared to mice on the 2018 control diet (35.2 g; p < 0.001).

#### Protein-malnutrition recovery

During the protein-malnutrition recovery phase, all intervention diets performed equally regarding weight gain (Fig 2A and 2C). During the 6-week period, mice in the control group increased their body weight by 28%, mice in the milk group by 32%, in the peanut group by 39% and in the cricket group by 34% (Fig 2C). These differences were not statistically significant. However, during the 6-week recovery period, malnourished animals did not reach the body weight of the control group (45.5 g by the end of the study) and remained significantly smaller with average weight of 38.7 g (p <0.0001; Fig 2A).

### Triglycerides, leptin and adiponectin

#### Protein-malnutrition

**Fig 3:**
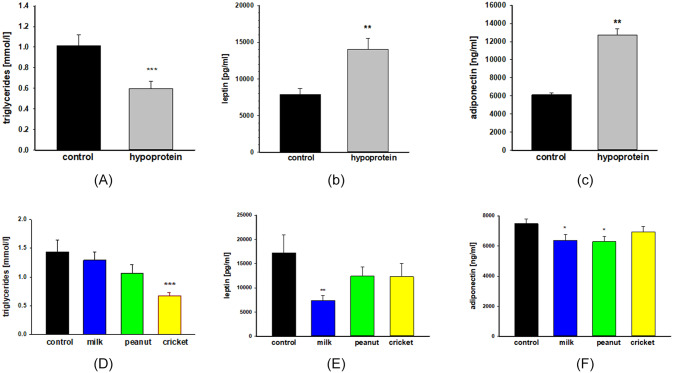
Metabolic markers. Serum levels of triglycerides **(A, D)**, leptin **(B, E)** and adiponectin **(C, F)** after 2 weeks of protein malnutrition **(A-C)** and after 6 weeks of the recovery period **(D-F)**. Triglyceride were analyzed using EnzyChrom Triglyceride Assay kit. Leptin and adiponectin were assayed using Quantikine ELISAs. One-way ANOVA analysis was used to determine difference between experimental groups (hypoprotein, milk, peanut, and cricket diets) and controls group (2018 diet). Results are expressed as means ± SE. n = 10-12/group, * p< 0.05, **p< 0.01, ***p< 0.001.

Analyses of samples taken after two weeks of hypoprotein diet displayed significant shifts in select metabolic biomarkers including triglycerides and adipokines. There was a 41% decrease in serum triglyceride levels (1.01 vs 0.59 mmol/l; p< 0.003) (Fig 3A). At the same time, serum leptin was increased by 78% (7885 vs 14042 pg/ml; p< 0.0032) and adiponectin was increased by 106% (6169 vs 12752 ng/ml; p< 0.001) compared to control mice (Fig 3B).

#### Protein-malnutrition recovery

Among samples analyzed after the six-week recovery period, the serum levels of the same metabolic biomarkers were also differentially regulated depending on recovery diet (Fig 3D, 3E and 3F). When compared to the control diet, levels of adiponectin were lower for mice on the milk diet (by 15%, p < 0.001) and the peanut diet (by 16%, p < 0.001). Mice on the milk diet also had lower levels of leptin compared to the control diet (by 57%, p <0.01). For triglycerides, only mice on the cricket diet had lower levels than controls (by 47%, p < 0.001).

### Markers of inflammation

#### Protein-malnutrition

**Fig 4:**
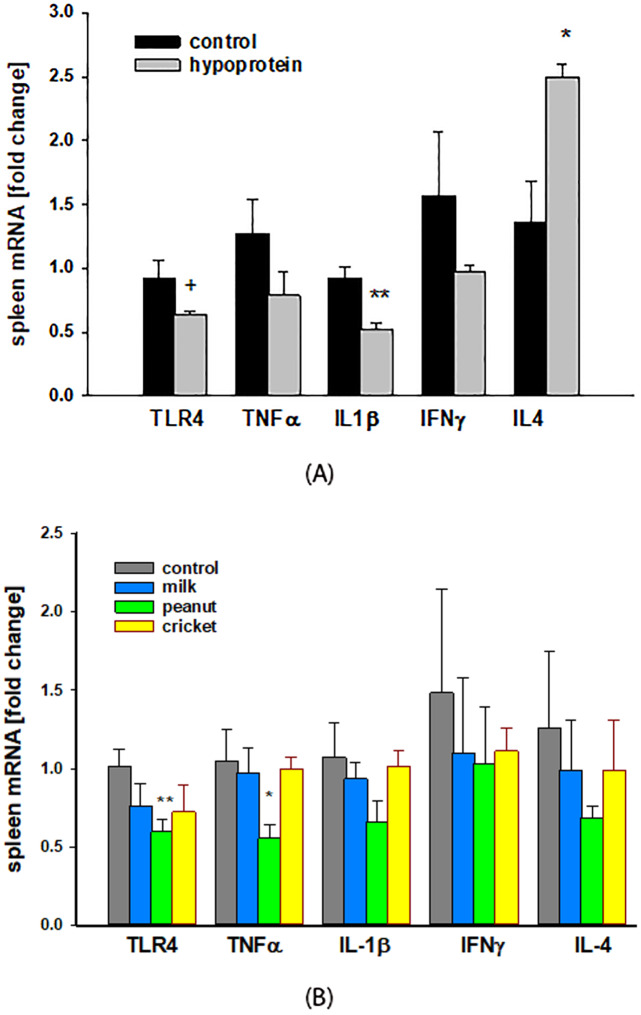
Inflammatory markers. Spleen gene expression assessed by quantitative PCR after two weeks of malnutrition induced by hypoprotein diet **(A)** and after 6-weeks recovery on the intervention diets **(B)**. One-way ANOVA analysis was used to determine difference between experimental groups (hypoprotein, milk, peanut, and cricket diets) and controls group (2018 diet). Results are expressed as means ± SE. n = 4–5 /group, + p = 0.056, * p≤ 0.05, **p< 0.01.

Fig 4A. presents the effects of acute protein-malnutrition induced with a two-week hypoprotein diet on the expression of several inflammatory (TLR4, TNFα, IL-1β, IFNγ) and anti-inflammatory (IL-4) markers in spleen tissue. TLR4 was downregulated in malnourished mice by 32% (p = 0.056). The pro-inflammatory gene IL-1β was also decreased by 44% (p< 0.002). The mRNA of two additional pro-inflammatory genes TNFα and INFγ was also downregulated (by 22% and 43%, respectively) although these differences did not reach statistical significance. Finally, mRNA levels of the anti-inflammatory cytokine IL-4 were significantly upregulated by 1.82-fold (p < 0.01).

#### Protein-malnutrition recovery

After six weeks on recovery protein diets, there were no differences observed between the expression of select inflammatory genes in the spleen among control group and the cricket or milk diet recovery groups (Fig 4B). However, mice on the peanut diet had a significantly higher expression of TLR4 (by 41%, p< 0.01) and TNFα(by 47%, *p* < 0.04) compared to control group.

## Discussion

In this study we investigated the effectiveness of a cricket-based diet on body weight and select metabolic and inflammatory markers in the recovery from protein malnutrition and compared it to peanut- and milk-based diets. Findings showed that the cricket diet performed similarly, but not exactly the same, as more traditional sources of proteins in recovery from malnutrition. As a dense source of micronutrients, vitamins and fatty acids, edible insects, in this instance crickets, may offer a viable alternative to protein sources that are traditionally used to treat malnutrition [37, 38]. Furthermore, crickets alongside other types of edible insects, including palm weevil larvae and termites, can provide a socially acceptable food source for communities, women and children who are at a high risk of undernutrition [29, 39, 40]. In this context, edible insects may provide a novel approach to securing adequate food resources, while offering an opportunity to combat unsustainable food production made worse by the growing pressures of climate change [10].

### The impact of protein malnutrition

Protein-malnutrition was induced by an isocaloric low protein diet (5% protein calories) in young male mice during puberty for two weeks followed by a six-week supplemental protein diet intervention. Body weight gain was a primary outcome of interest throughout the study because it is representative of normal growth and development traditionally used in studies of chronic malnutrition. Administration of the hypoprotein diet for two weeks resulted in stunted growth; however, the malnutrition was not severe enough to cause body weight loss or wasting. Mice on the control diet gained on average 6.2 g of body weight during this period whereas mice on the hypoprotein diet gained only 0.7 g. Puberty is an important developmental stage accompanied by hormonal and physiological changes; therefore, malnutrition during this period may have lasting effects on brain development, sexual maturation, immune function and metabolism [41, 42].

Protein-malnutrition is often associated with dysregulated energy homeostasis and metabolism including altered blood lipid levels [43–45]. In this study, protein-malnutrition resulted in decreased levels of serum triglycerides likely due to lower availability of dietary fats, a finding that was observed in other animal and clinical studies [44, 46]. We also examined the impact of protein-malnutrition on two adipokines, leptin and adiponectin, produced by adipose tissue that have pleiotropic effects from regulation of energy metabolism, energy storage and food intake to hematopoiesis, modulation of the immune system and sexual maturation [47]. We observed elevated blood levels of adiponectin, a finding consistent with a prior study in suckling in rats [48]. On the other hand, the elevated blood levels of leptin during malnutrition was an intriguing observation, since leptin production is usually proportional to body fat mass while adiponectin levels are usually inversely correlated with adiposity [49–52]. In this study, we measured weight gain but not body composition. It is possible that while the hypoprotein diet resulted in decreased weight gain, it may have contributed to altered body composition including higher body fat percentage and thus increased leptin levels, however, further investigations are warranted. Previous studies showed that a low protein diet may increase total food intake in order to satisfy protein requirements, subsequently resulting in an increased body fat percentage [53–55]. Additionally, it is possible that elevated levels of leptin observed in our study might have been due to a higher proportion of calories (84%) from carbohydrates in the hypoprotein diet compared to 60–65%carbohydrate content in regular diet as some studies found that high carbohydrate diet is associated with an increase of leptin levels in humans [56]. However, the existing literature on leptin levels during malnutrition is mixed. Studies of early postnatal protein-malnutrition in rats did not find changes in blood leptin levels [48], whereas protein-malnutrition in young adult rats was associated with higher leptin levels [53]. Another study showed higher leptin levels in early postnatal malnutrition whereas malnutrition in two-month old rats was associated with decreased leptin blood levels [57]. Therefore, it is likely that metabolic adaptation, including adipokine production, will depend on the severity and duration of malnutrition as well as age when malnutrition is experienced. Nevertheless, our results suggest a potential dysregulation of adipokine production associated with protein-malnutrition in pubertal male mice. More studies are needed to better understand the role and regulation of adipokines in protein-malnutrition together with short and long-term consequences on metabolism and immunity.

Altered immune function is also a serious, life threatening consequence of malnutrition in childhood that leads to increased morbidity and mortality from infectious diseases. Our findings of downregulated pro-inflammatory genes and upregulated anti-inflammatory genes in spleen during protein-malnutrition are consistent with other studies [58, 59]. Among the genes we evaluated was Toll-like receptor 4 (TLR4) belonging to a Toll-like receptors superfamily of pathogen recognition receptors. TLRs are membrane proteins expressed by innate immune cells that detect components of viruses, bacteria, fungus and protozoa [51]. Activation of TLRs leads to production of cytokines and chemokines that orchestrate innate and adaptive immune responses to infections [60]. As a gram-negative bacteria sensor, downregulation of TLR4 during malnutrition suggests decreased ability to recognize and respond to infections, including those caused by *Escherichia coli*. This idea is supported by a study of protein-malnutrition in adult mice that resulted in decreased expression of TLR4 in macrophages and deficient response to Lipopolysaccharide (LPS), a lipoglycan from *E*. *Coli* [58]. Other studies also observed impaired immune responses in malnutrition [61].

### Recovery from protein malnutrition

Similar to growth-retardation during malnutrition, body weight recovery is an important indicator in determining the efficacy of supplemental foods used to treat malnutrition early in life [15, 62]. Underweight status is a primary global indicator of mortality in children under five [63]. Recovery diets, after chronic malnutrition, therefore, should meet the needs for growth and development [17]. We show here that for body weight recovery, the cricket-based diet performed equally well when compared to the peanut- and milk-based diets. Body weight increases during protein-malnutrition recovery were equal across all recovery diets, which were also comparable to the control diet. However, after six weeks of recovery, mice fed the cricket-, peanut- and milk-based diets remained smaller than mice fed the control diet who were never exposed to protein-malnutrition. A prior study in protein-malnourished rats observed that palm weevil- and cricket-based diets performed equally well to a control diet when assessing weight gain, bone mineral content, lean and fat mass, and organ weights [32]. Additionally, diets fortified with spiders and termites improved weight gain among infants living in resource poor regions [64]. Beyond adequate protein and protein quality for weight gain, sufficient amounts of macro- and micronutrients and energy, are all important and necessary components for treatment diets to combat malnutrition [17]. In this regard, insect diets, in particular crickets may be a suitable and sustainable complete protein source because they are not only able to support increased weight gain during recovery as shown in this study, but previous investigations indicate they also contain adequate amounts of all essential amino acids to meet treatment diet requirements.

We observed differential effect of the cricket diet on triglyceride levels compared to the milk and peanut diets. After the recovery period, mice fed the milk and peanut diets had triglyceride levels comparable to controls, whereas triglyceride levels remained significantly lower in mice fed the cricket diet despite the fact that the percent of calories from total fat were greater in the cricket diet (24%) than the milk (20%) or peanut (19%) diets. It is possible that lower triglyceride levels following the cricket diet may have a positive health impact. Elevated triglyceride levels can be a risk factor for poor cardiovascular health outcomes [65], and there is currently no minimum recommendation for triglyceride levels. Even after recovery, malnutrition experienced during childhood is linked to increased risk of non-communicable diseases including diabetes, obesity and cardiovascular diseases in adulthood [7]. Therefore, further longitudinal investigations should be designed to determine whether treating malnutrition using crickets or other edible insects could decrease the risk of obesity or cardiovascular morbidity into adulthood. There were also differences across the recovery diets when considering blood levels of adiponectin and leptin. Several studies showed a significant association between serum levels of leucine and isoleucine, branched-chain amino acid (BCAA), with serum triglyceride and adipokines that play a significant role in development of insulin resistance and type 2 diabetes [66–68]. The recovery diets used in this study have likely different amino acid composition including levels of BCCAs. Crickets provide a complete protein containing all essential amino acids. Milk is also a rich source of BCAAs whereas wheat protein, the main protein source in control diet, contains lower levels of BCAAs. We did not measure BCAA levels in diets or serum levels of free BCAAs in this study, but such studies are needed to provide insights into the relationship between dietary amino acids and metabolic outcomes and specifically the role of BCAAs in the recovery from malnutrition.

The analysis of gene expression in spleen tissue revealed that mice on the cricket- and milk-based diets had similar expression of inflammatory genes when compared to mice fed the control diet. On the other hand, the expression of TLR4 and TNFα remained lower in mice fed the peanut-based diet, which suggests greater immune response dysfunction for this group.

### Conclusions

Study findings provide important advances in the current knowledge regarding insects as one potential option to combat global protein malnutrition, a significant and persistent cause of global morbidity and mortality. Protein malnutrition is not only detrimental for child development, it also has lasting health effects and increases population vulnerability and susceptibility to acute and chronic disease across the life-course. In order to meet the important balance between nutritional needs and food system sustainability, alternative protein sources in addition to conventional livestock may be needed. Findings add to the growing body of evidence in support of edible insects and, more specifically, cricket protein as another possible option available to combat the effects of chronic malnutrition in early-life.

## Supporting information

S1 File (XLSX)

S2 File EnzyChrom triglyceride assay. (PDF)

S3 File Leptin Quantikine ELISA. (PDF)

S4 File Adiponectin Quantikine ELISA. (PDF)

S5 File Gene expression, fold change hypoprotein (HP) diet vs 2020 diet. (PDF)

S6 File Gene expression; end of recovery period. (PDF)

## Abbreviations

ELISA: enzyme-linked immunosorbent assay
IL-4: interleukin 4
IL-1β: interleukin 1b
INFγ: interferon gamma
TLR4: toll-like receptor 4
TNFα: tumor necrosis factor alpha
