# Supplementary figures and images for: Comparison of cricket diet with peanut-based and milk-based diets in the recovery from protein malnutrition in mice and the impact on growth, metabolism and immune function
Source: PLoS One. 2020 Jun 11;15(6):e0234559. doi: 10.1371/journal.pone.0234559 (PMC7289377; doi:10.1371/journal.pone.0234559)

S2: EnzyChrom Triglyceride Assay

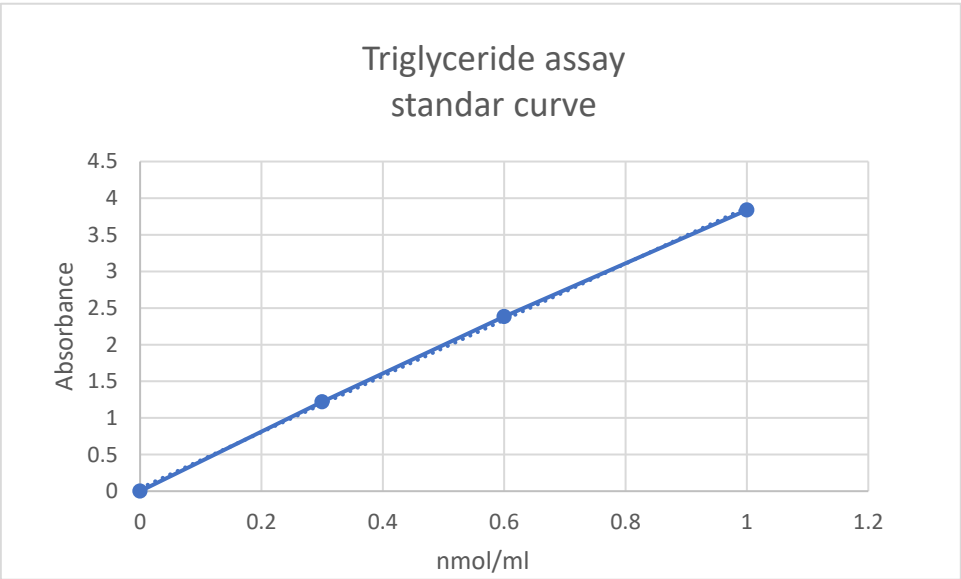

| Diet   | 2020   | hypoprotein | 2018   | cricket | milk   | peanut |
|--------|--------|-------------|--------|---------|--------|--------|
| mmol/l | 1.2460 | 1.3653      | 0.5522 | 0.8815  | 0.7224 | 0.7615 |
|        | 1.6085 | 0.6937      | 2.5494 | 0.5913  | 1.7278 | 0.4889 |
|        | 1.2075 | 0.5176      | 0.8854 | 0.6767  | 1.1651 | 2.1328 |
|        | 0.6350 | 0.4531      | 1.8002 | 0.3676  | 1.2525 | 1.3014 |
|        | 0.8378 | 0.4948      | 1.0901 | 0.6004  | 0.7986 | 1.5400 |
|        | 0.6030 | 0.3918      | 2.0317 | 1.0758  | 1.9906 | 0.7061 |
|        | 0.6135 | 0.5443      | 1.2381 | 0.7778  | 1.1957 | 0.9056 |
|        | 1.0686 | 0.4198      | 0.7973 | 0.5059  | 0.8886 | 1.4690 |
|        | 1.0947 | 0.6343      | 1.5081 | 0.5743  | 1.7774 | 0.4087 |
|        | 1.2147 | 0.5828      | 1.9085 | 0.4993  | 1.4018 | 0.9734 |
|        |        | 0.4870      |        | 0.8006  |        | 0.9890 |
|        |        | 0.5730      |        | 0.7184  |        |        |

Supplement: S2 File — (PDF) [file pone.0234559.s002.pdf]

S3: Leptin Quantikine ELISA

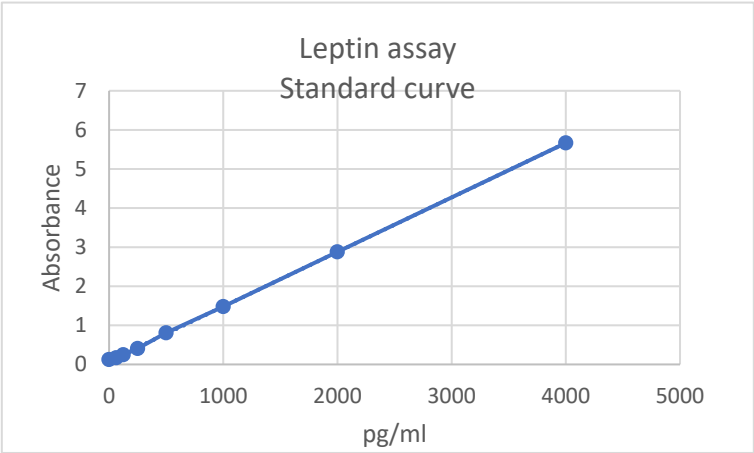

| Diet  | 2020     | hypoprotein | 2018     | cricket  | milk     | peanut   |
|-------|----------|-------------|----------|----------|----------|----------|
| pg/ml | 3049.86  | 19412.24    | 2540.33  | 21292.24 | 11150.81 | 2784.62  |
|       | 6506.05  | 15957.00    | 14668.90 | 7820.81  | 6132.24  | 8143.19  |
|       | 7876.05  | 13671.76    | 6247.95  | 9812.24  | 6560.81  | 17274.14 |
|       | 5345.10  | 10814.62    | 36456.52 | 3793.19  | 8537.48  | 16919.86 |
|       | 8793.67  | 10757.00    | 16030.81 | 10091.29 | 5347.95  | 3401.76  |
|       | 10417.95 | 1504.00     | 11678.90 | 36486.05 | 12551.29 | 14569.38 |
|       | 9155.10  | 18363.67    | 19567.48 | 11374.62 | 6136.52  | 21462.71 |
|       | 7055.57  | 14590.81    | 15754.62 | 10667.95 | 1887.48  | 15215.10 |
|       | 8546.52  | 14322.24    | 32400.81 | 2861.29  | 5219.38  | 12783.67 |
|       | 12110.81 | 17214.14    |          | 3774.62  | 10132.71 | 11544.62 |
|       |          | 11848.90    |          | 13688.43 |          |          |
|       |          | 20050.33    |          | 16151.76 |          |          |

Supplement: S3 File — (PDF) [file pone.0234559.s003.pdf]

#### S4: Adiponectin Quantikine ELISA

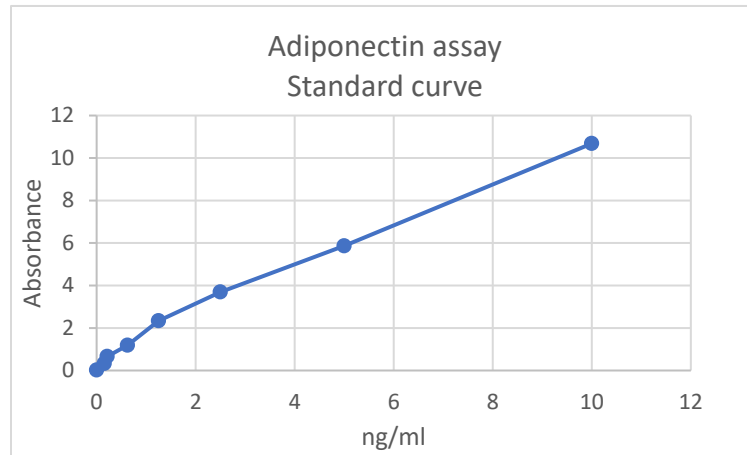

| Diet  | 2020    | hypoprotein | 2018    | cricket | milk    | peanut  |
|-------|---------|-------------|---------|---------|---------|---------|
| ng/ml | 6032.95 | 15480.38    | 7320.91 | 8029.55 | 8435.70 | 8124.42 |
|       | 5765.87 | 11807.47    | 7060.42 | 6611.25 | 7748.64 | 6388.47 |
|       | 6306.88 | 15444.03    | 7152.33 | 5490.58 | 6585.75 | 7014.58 |
|       | 6666.26 | 13204.97    | 8768.80 | 4983.14 | 4676.36 | 6046.38 |
|       | 6542.65 | 9024.42     | 6011.86 | 7375.31 | 5346.75 | 7274.66 |
|       | 5956.33 | 12916.83    | 8433.62 | 8242.37 | 6662.33 | 3994.38 |
|       | 4326.74 | 10705.72    | 6607.33 | 7939.06 | 6235.21 | 6252.63 |
|       | 6780.56 | 13067.67    | 6326.28 | 7136.32 | 4866.39 | 4997.78 |
|       | 5895.22 | 11271.17    | 8739.34 | 4781.10 | 6833.93 | 7080.37 |
|       | 6994.67 | 12354.48    | 8412.78 | 7885.71 | 6441.07 | 6411.83 |
|       |         | 17254.54    |         | 6764.76 |         | 5737.43 |
|       |         | 10493.91    |         | 8021.31 |         |         |

Supplement: S4 File — (PDF) [file pone.0234559.s004.pdf]
