# Supplementary material for: Comparison of cricket diet with peanut-based and milk-based diets in the recovery from protein malnutrition in mice and the impact on growth, metabolism and immune function
Source: PLoS One. 2020 Jun 11;15(6):e0234559. doi: 10.1371/journal.pone.0234559 (PMC7289377; doi:10.1371/journal.pone.0234559)
Supplement: S5 File — (PDF) [file pone.0234559.s005.pdf]

S5: Gene expression, fold change

hypoprotein (HP) diet vs 2020 diet

TLR4		TNFα		IL1-β		IFNγ		IL4	
2020	HP	2020	HP	2020	HP	2020	HP	2020	HP
0.98	0.61	0.83	1.44	0.98	0.49	0.40	1.08	2.44	2.77
1.31	0.78	0.88	0.83	1.08	0.67	0.85	0.95	0.29	2.12
0.73	0.68	0.94	0.65	0.86	0.69	3.11	1.00	0.96	2.62
0.86	0.57	1.21	0.43	0.99	0.36	1.16	0.79	1.28	2.48
1.23	0.56	1.20	0.59	1.11	0.42	0.81	1.01	1.14	2.46
0.44	0.60	2.56		0.54	0.49	3.09		2.06	
