# Supplementary material for: Comparison of cricket diet with peanut-based and milk-based diets in the recovery from protein malnutrition in mice and the impact on growth, metabolism and immune function
Source: PLoS One. 2020 Jun 11;15(6):e0234559. doi: 10.1371/journal.pone.0234559 (PMC7289377; doi:10.1371/journal.pone.0234559)
Supplement: S6 File — (PDF) [file pone.0234559.s006.pdf]

S6: Gene expression; end of recovery period

**TLR4**			
2018	cricket	milk	peanut
1.33	0.04	1.19	0.76
0.93	1.15	0.60	0.60
0.86	0.98	0.69	0.75
0.95	1.04	0.56	0.57
	0.50		0.31
	0.64		
**TNFα**			
2018	cricket	milk	peanut
0.75	1.14	1.36	0.75
0.82	1.05	0.70	0.56
1.00	1.20	1.09	0.65
1.63	0.73	0.75	0.57
	0.87		0.26
	1.01		
**IL1β**			
2018	cricket	milk	peanut
0.65	1.18	1.15	1.11
1.40	1.15	0.87	0.49
0.73	1.11	1.04	0.83
1.50	1.01	0.67	0.37
	0.56		0.52
	1.10		
**IFNγ**			
2018	cricket	milk	peanut
0.25	1.25	0.95	0.86
3.24	0.70	0.52	0.50
0.70	0.85	2.50	0.86
1.75	0.97	0.44	0.52
	1.19		2.43
	1.70		
**IL4**			
2018	cricket	milk	peanut
0.47	0.86	0.71	0.64
2.62	1.82	0.98	0.43
0.63	0.31	0.40	0.77
1.30	1.38	1.87	0.91
	0.54		0.67
	0.63		
